# Analysis of the regulation of undecaprenyl diphosphate dephosphorylation in *Escherichia coli*

**DOI:** 10.1002/2211-5463.70066

**Published:** 2025-06-13

**Authors:** Tomotaka Jitsukawa, Yasushi Shigeri, Shingo Fujisaki

**Affiliations:** ^1^ Department of Biomolecular Science, Faculty of Science Toho University Funabashi Japan; ^2^ Department of Chemistry Wakayama Medical University Japan

**Keywords:** bacitracin, *Escherichia coli*, undecaprenyl diphosphate, undecaprenyl phosphate

## Abstract

Undecaprenyl phosphate (C_55_P) is an essential sugar carrier for bacterial cell wall synthesis, which has gained importance in recent years as a promising target for new antibiotic development. In *Escherichia coli*, C_55_P is produced by dephosphorylation of undecaprenyl diphosphate (C_55_PP) by BacA and two type 2 phosphatidic acid phosphatase (PAP2) family enzymes, PgpB and YbjG, in the periplasmic space. To clarify the regulatory mechanism of C_55_PP dephosphorylation, we quantified C_55_P and C_55_PP using a new high‐performance liquid chromatography method, conducted susceptibility tests against bacitracin, and analyzed the gene expression of *bacA*, *pgpB*, and *ybjG* in *E. coli* single‐ and double‐disruption strains of those genes. C_55_P levels were similar in all strains, but C_55_PP levels increased only in the *bacA, ybjG* double‐disruption strain. The double‐disruption strains containing *bacA* disruption and the *bacA* single‐disruption strain were more susceptible to bacitracin than the other strains. In the double‐disruption strains containing *bacA* disruption, the expression of the remaining genes *pgpB* and *ybjG* increased. These results indicate that the transcription of the PAP2 family enzyme genes, *pgpB* and *ybjG*, was activated under conditions where C_55_PP dephosphorylation activity in cells was reduced. This transcriptional regulation might contribute to the maintenance of C_55_P levels in cells.

## Graphical abstract

BacA, PgpB, and YbjG phosphatases are involved in undecaprenyl phosphate (C_55_P) synthesis in *Escherichia coli*. We analyzed the lipid contents and the gene expression in the gene‐disruption strains. Undecaprenyl diphosphate (C_55_PP) level increased in the *bacA, ybjG* double‐disruption strain, but C_55_P levels were similar in all strains. In the *bacA‐*deficient strains, residual gene expression increased, suggesting mechanisms to maintain C_55_P levels.

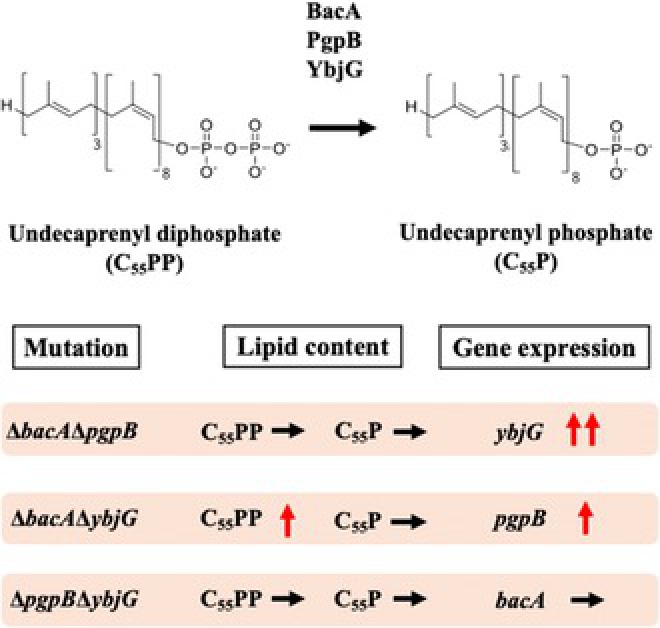

**Abbreviations**
C_45_P: nonaprenyl phosphate
C_45_PP: nonaprenyl diphosphate
C_55_P: undecaprenyl phosphate
C_55_PP: undecaprenyl diphosphate
ECA: enterobacterial common antigen
GlcNAc: *N*‐acetyl glucosamine
HPLC: high‐performance liquid chromatography
KPB: potassium phosphate buffer
MurNAc: *N*‐acetyl muramic acid
OD_600_
: optical density at 600 nm
PAP2: type 2 phosphatidic acid phosphatase
UDP: uridine diphosphate

Undecaprenyl phosphate (C_55_P) functions as a sugar carrier in the biosynthesis of bacterial cell‐envelope polysaccharides such as peptidoglycan [1], O‐antigen [2], and enterobacterial common antigen [3] (Fig. 1A) and is an essential molecule for bacterial survival [4, 5, 6]. In recent years, as the problem of increasing drug resistance in bacteria has become more serious, the biosynthetic pathway of C_55_P is being considered as a promising target for new antibiotics [4, 7]. In the biosynthetic pathway of C_55_P, undecaprenyl diphosphate (C_55_PP) is synthesized in the cytoplasm by the sequential condensation of eight molecules of isopentenyl diphosphate with farnesyl diphosphate by the action of C_55_PP synthase (IspU/Rth) [8, 9] (Fig. 1B). C_55_P is then produced by the dephosphorylation of C_55_PP. Uridine diphosphate sugars react sequentially with C_55_P to form C_55_PP‐linked sugar intermediates for polysaccharide biosynthesis [1, 2, 3]. The C_55_PP‐linked sugar intermediates are transported to the outside of the cytoplasmic membrane by specific flippases and become substrates for polysaccharide biosynthesis [10, 11]. C_55_PP is released into the periplasmic leaflet during polysaccharide synthesis [1, 2, 3]. C_55_PP is dephosphorylated to form C_55_P [12], which is then transported to the inside of the cytoplasmic membrane [13, 14]. This is called the recycling pathway for C_55_P synthesis [4].

**Fig. 1:**
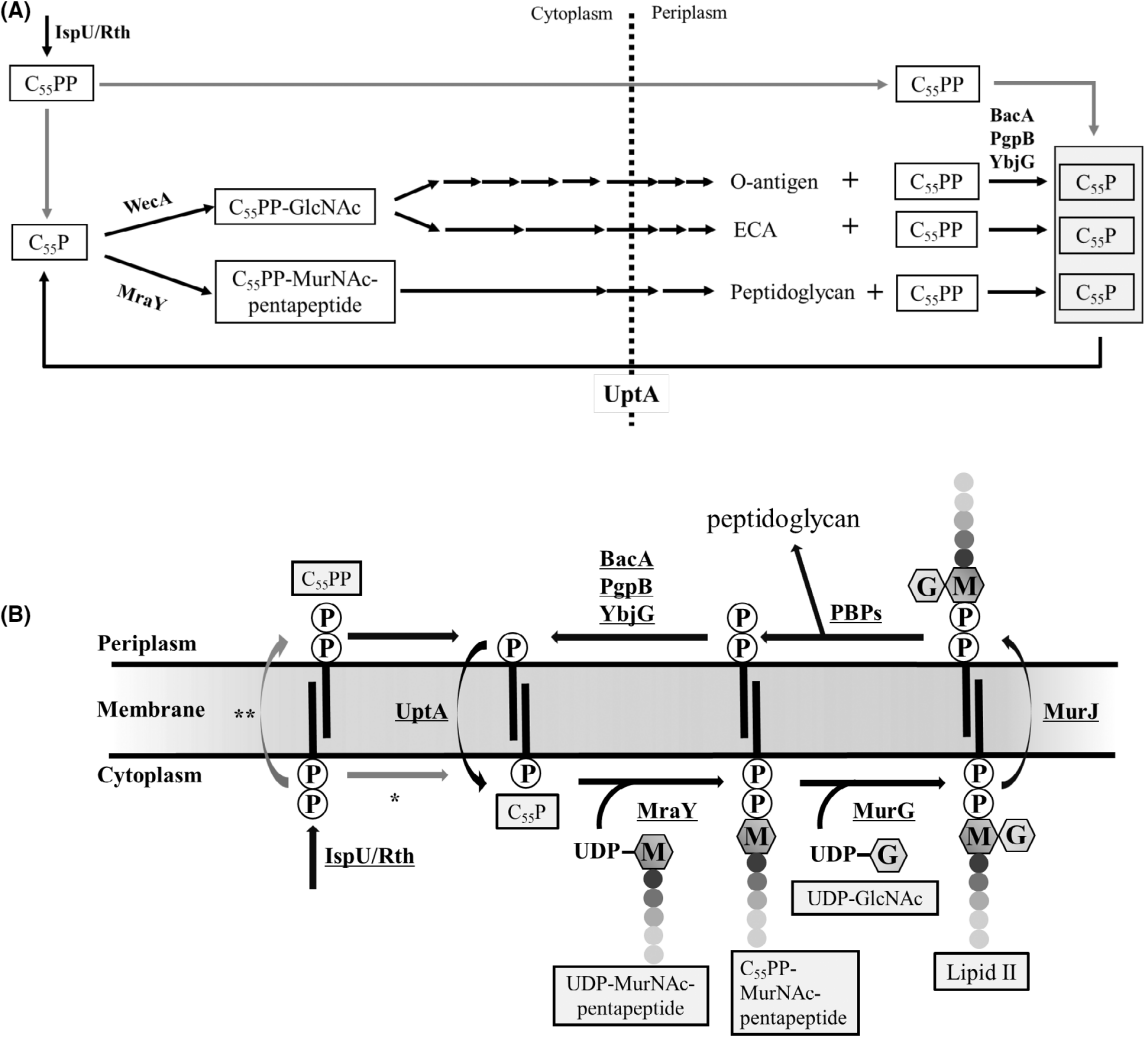
Function of C_55_P in *Escherichia coli*. (A) Schematic representation of the role of C_55_P in the biosynthesis of peptidoglycan, O‐antigen, and enterobacterial common antigen (ECA). Peptidoglycan biosynthesis is initiated by the enzymatic action of MraY, while O‐antigen and ECA biosynthesis are initiated by the enzymatic action of WecA. After each multistep enzymatic reaction, the generated compounds are transported to the outside of the cytoplasmic membrane by specific transporters. The arrows indicate enzymatic reactions and actions of transporters. (B) The function of C_55_P in the peptidoglycan biosynthetic pathway. The short black bar represents an undecaprenyl group, the circled P represents a phosphate group, the capital letter “M” surrounded by a hexagon represents an *N*‐acetyl muramic acid (MurNAc), and the capital letter “G” surrounded by a hexagon represents an *N*‐acetyl glucosamine (GlcNAc). At the inside of the cytoplasmic membrane, uridine diphosphate (UDP)‐MurNAc pentapeptide and UDP‐GlcNAc are condensed with C_55_P, reactions catalyzed by MraY and MurG, respectively, to generate lipid II. The generated lipid II is transported from the inside to the outside of the cytoplasmic membrane by MurJ. The disaccharide pentapeptide is incorporated into peptidoglycan chains by penicillin‐binding proteins. The released C_55_PP is dephosphorylated by BacA, PgpB, and YbjG to generate C_55_P. C_55_P is transported from the outside to the inside by UptA and then recycled. In the *de novo* synthesis, C_55_PP is synthesized in the cytoplasm. There are two possibilities for the *de novo* synthesis of C_55_P. The first one is the dephosphorylation of C_55_PP at the cytoplasmic side by an unidentified C_55_PP phosphatase that acts inside of the cytoplasmic membrane (*). The second one is the dephosphorylation of C_55_PP by BacA or PAP2 enzyme after the translocation of C_55_PP from the inside to the outside of the cytoplasmic membrane (**). The latter mechanism requires the flippases that enhance the translocation of C_55_PP and C_55_P across the cytoplasmic membrane.

In many bacteria, several enzymes are involved in the dephosphorylation of C_55_PP in the periplasmic space [4, 5, 6] (Fig. 1B). Specifically, a highly substrate‐specific C_55_PP phosphatase belonging to the BacA family and some relatively low substrate‐specific lipid phosphatases belonging to the type 2 phosphatidic acid phosphatase (PAP2) family are involved in this step [15, 16]. In *Escherichia coli*, BacA and two lipid phosphatases belonging to the PAP2 family, PgpB and YbjG, have been confirmed to be involved in the dephosphorylation of C_55_PP [15]. Another PAP2 enzyme, LpxT, transfers phosphate from C_55_PP to lipid A *in vivo*, although it exhibits activity that hydrolyzes C_55_PP *in vitro* [17]. Single‐ and double‐deletion mutations of *bacA*, *pgpB*, and *ybjG* do not affect the growth rate, but the triple deletion of *bacA*, *pgpB*, and *ybjG* is lethal, indicating that these genes share redundant roles in the essential periplasmic C_55_PP dephosphorylation activities [15]. By comparing the C_55_PP phosphatase activity of the membrane fraction of the *bacA* disruption strain with that of the parental strain, it is known that BacA is the major C_55_PP phosphatase, accounting for approximately 75% of the total C_55_PP phosphatase activity [12]. By amplifying the *bacA* gene, *E. coli* becomes more resistant to the antibiotic bacitracin, which inhibits the dephosphorylation of C_55_PP by binding to it [18, 19].

BacA [20, 21] and all PAP2 enzymes [22, 23, 24] are localized in the cytoplasmic membrane, with their active sites located on the periplasmic side. In the *de novo* synthesis of C_55_P, the site of the dephosphorylation of C_55_PP synthesized on the inner side of the cytoplasmic membrane [8, 9] remains unresolved (Fig. 1B). The phosphatase responsible for the dephosphorylation of C_55_PP inside the cytoplasmic membrane is currently unknown. Alternatively, C_55_PP may be transported to the periplasmic side by unknown flippases and then dephosphorylated by phosphatases involved in the recycling pathway, such as BacA, PgpB, and YbjG.

The availability of C_55_P is important for normal bacterial growth [25, 26]. Since several phosphatases are involved in the dephosphorylation of C_55_PP, there may be a regulatory mechanism for their enzymatic activity. However, the extent to which the three phosphatases contribute to the dephosphorylation of C_55_PP in living *E. coli* cells is not known, nor is the regulatory mechanism. To determine whether dephosphorylation of C_55_PP is the rate‐limiting step in the supply of C_55_P *in vivo*, it is necessary to measure the levels of C_55_P and C_55_PP simultaneously in cells. We have developed a new method to quantify polyprenyl phosphates and polyprenyl diphosphates by extracting them from cells and then analyzing them by high‐performance liquid chromatography (HPLC) [27]. To elucidate the regulation of C_55_PP dephosphorylation activity, we generated single‐ and double‐disruption strains of the *E. coli* phosphatase genes [28] and measured the levels of C_55_P, C_55_PP, and total C_55_P derivatives. In addition, we performed a susceptibility test to bacitracin as an indicator of reduced C_55_PP dephosphorylation activity. Finally, we examined changes in the expression levels of C_55_P synthesis‐related genes in the gene‐disruption strains.

## Materials and methods

### Chemicals

Nonaprenyl phosphate (C_45_P) and nonaprenyl diphosphate (C_45_PP) were synthesized by phosphorylation of commercial nonaprenol (Tokyo Chemical Industry, Tokyo, Japan) as previously described [27].

### Bacterial strains and growth conditions

**Fig. 2:**
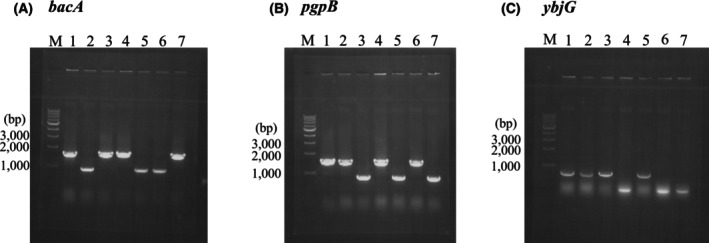
Construction of the gene‐disruption strains. Colony PCR analysis of the chromosome of the gene‐disruption strains and the parental strain was carried out twice, and representative results are shown here. DNA fragments of the *bacA* (A), *pgpB* (B), and *ybjG* (C) were amplified and separated by electrophoresis in a 0.85% agarose gel. Lanes 1, W3110; 2, W3110Δ*bacA*; 3, W3110Δ*pgpB*; 4, W3110Δ*ybjG*; 5, W3110Δ*bacA*Δ*pgpB*; 6, W3110Δ*bacA*Δ*ybjG*; 7, W3110Δ*pgpB*Δ*ybjG*; M, 1 kb DNA ladder.

The bacterial strains used in this study are listed in Table 1. Strains JWK1270, JWK3029, and JWK5112 [29], and a plasmid pCP20 [30] were provided by the National BioResource Project (NIG, Mishima, Shizuoka, Japan): *E. coli*. Single‐ and double‐gene‐disruption strains of *E. coli* were constructed by transduction with P1 phage and transformation with pCP20 as previously reported [28, 30]. Gene disruption was confirmed by performing colony PCR (Fig. 2) using primers listed in Table 2. *E. coli* was grown in L‐broth (10 g polypeptone, 5 g yeast extract, 5 g NaCl, 1 g glucose, and 4.25 mmol NaOH in 1000 mL H_2_O).

**Table 1:** Bacterial strains.

Strain	Genotype	References
JWK3029	BW25113Δ*bacA*::Km	[29]
JWK1270	BW25113Δ*pgpB*::Km	[29]
JWK5112	BW25113Δ*ybjG*::Km	[29]
W3110	Parental strain	Laboratory stock
W3110Δ*bacA*	W3110, Δ*bacA*	[28]
W3110Δ*pgpB*	W3110, Δ*pgpB*	This work
W3110Δ*ybjG*	W3110, Δ*ybjG*	This work
W3110Δ*bacA*Δ*pgpB*	W3110, Δ*bacA*Δ*pgpB*	This work
W3110Δ*bacA*Δ*ybjG*	W3110, Δ*bacA*Δ*ybjG*	This work
W3110Δ*pgpB*Δ*ybjG*	W3110, Δ*pgpB*Δ*ybjG*	This work

**Table 2:** Oligonucleotides used for colony PCR.

Gene	Primers	Sequences (5′–3′)	References
*bacA*	Fw	CTGCTCCCTTGCCACCGATA	[28]
	Rv	AGCGTCGCATCAGGCGTTGA	[28]
*pgpB*	Fw	ACAAGCGGACTTCATTGACG	[28]
	Rv	TATGGTCAACTTACCGCAAT	[28]
*ybjG*	Fw	CTTGAGGGAAATAAGACGAT	[28]
	Rv	CTCTGAATAGTTATTGAAGCGA	[28]

### Extraction and purification of polyprenyl phosphates and diphosphates from *E. coli*

The extraction and purification of polyprenyl phosphates and diphosphates from *E. coli* was carried out as described previously [27]. Cells were cultured in L‐broth for 3 h with shaking at 37 °C. After measuring optical density at 600 nm (OD_600_) for cell density, cells grown in 20 mL of medium were collected and suspended in 1 mL of 0.1 m potassium phosphate buffer (KPB) (pH 7.4). Then, 2.5 mL of methanol and 1.25 mL of chloroform were added to the suspension. As internal standards for the quantification of C_55_P and C_55_PP, respectively, 4.47 μg of C_45_P and 2.37 μg of C_45_PP were added to the mixture. After mixing for 2 min, the mixture was allowed to stand at room temperature for 10 min. After centrifugation at 1500 ***g*** for 10 min, the supernatant was transferred to a new perfluoroalkoxy alkane test tube. To the supernatant, 1.25 mL of chloroform and 1.25 mL of 0.1 m KPB (pH 7.4) were added and then vortexed for 2 min. After standing at room temperature for 5 min and centrifuging at 1500 ***g*** for 5 min, the lower chloroform layer was collected. The chloroform extract was dried under nitrogen gas flow and dissolved in 1 mL of chloroform–methanol–water (10 : 10 : 3, v/v/v). The resulting solution was applied to a column packed with 0.5 g of Supelclean LC‐NH_2_ SPE (Merck, Darmstadt, Germany). After washing with 3 mL of chloroform–methanol–water (10 : 10 : 3, v/v/v), 4 mL of chloroform–methanol–water (2 : 0.9 : 0.1, v/v/v), and 4 mL of chloroform–methanol–water (2 : 0.9 : 0.1, v/v/v) containing 0.1 m ammonium acetate, polyprenyl phosphates were eluted with 10 mL of chloroform–methanol–water (2 : 0.9 : 0.1, v/v/v) containing 0.2 m ammonium acetate and then polyprenyl diphosphates were eluted with 10 mL of chloroform–methanol–water (10 : 10 : 3, v/v/v) containing 0.4 m ammonium acetate. Each of the eluents was washed with 1.5 mL of 0.1 m KPB (pH 7.4), and the chloroform layer was taken as the polyprenyl phosphate and diphosphate fraction.

### Extraction and purification of polyprenyl phosphates after alkaline hydrolysis

The extraction of total polyprenyl phosphate derivatives from *E. coli* was carried out as described previously [27]. Cells grown in 20 mL of medium were collected and suspended in 2 mL of methanol. After adding 3.87 μg of C_45_P as an internal standard for the quantification of total C_55_P derivatives, 1 mL of 60% KOH was added to the suspension and heated in a boiling water bath for 1 h. After cooling to room temperature, polyprenyl phosphates were extracted three times with 2 mL, 2 mL, and finally 3 mL of diethyl ether. The diethyl ether extracts were combined and washed with 2 mL of 5% acetic acid. The polyprenyl phosphates were purified by ion‐exchange chromatography as described above.

### HPLC analysis of polyprenyl phosphates and diphosphates

The analysis of polyprenyl phosphates and diphosphates by HPLC was carried out as described previously [27]. The polyprenyl phosphate and diphosphate fractions dried under nitrogen gas flow were dissolved in 300 and 100 μL of 25 mm aqueous tetraethylammonium phosphate (pH 7.5)–2‐propanol (35 : 65, v/v), respectively. The polyprenyl phosphate fractions were analyzed by reversed‐phase HPLC using a Mightysil RP‐18 GP Aqua column (5 μm, 150 × 4.6 mm) (Kanto Chemical, Tokyo, Japan). The mobile phase used was 2‐propanol–methanol (1 : 4, v/v) containing 5 mm phosphoric acid at a flow rate of 1.5 mL·min^−1^ and the column temperature was set at 40 °C. The polyprenyl diphosphate fractions were analyzed by reversed‐phase ion‐pair HPLC using a Cadenza CD‐C18 MF column (Imtakt, Kyoto, Japan). The mobile phase used was 25 mm aqueous tetraethylammonium phosphate (pH 7.5)–2‐propanol (2 : 3, v/v) at a flow rate of 0.15 mL·min^−1^ and the column temperature was set at 60 °C. Polyprenyl phosphates and diphosphates were detected at an absorbance of 210 nm and C_55_P and C_55_PP were quantified by the peak area ratio to the corresponding internal standard, C_45_P and C_45_PP, respectively.

### Bacitracin susceptibility

Cells were cultured in L‐broth with shaking at 37 °C overnight. The culture broth was normalized to OD_600_ = 1, and 10‐fold serial dilutions were made. Five microliter of each dilution was plated on L‐broth agar containing bacitracin (Toronto Research Chemicals, Vaughan, Ontario, Canada) at concentrations between 0.5 and 2 mg·mL^−1^. Plates were incubated at 37 °C for 24 h and photographed.

### RNA purification and cDNA synthesis

Cells were cultured in L‐broth for 3 h at 37 °C with shaking. To 1 mL of RNA Protect Bacterial Agent (Qiagen, Hilden, Germany), 500 μL of the culture broth was added and vortexed for 5 s. After standing at room temperature for 5 min, the mixture was centrifuged at 5000 ***g*** for 10 min, and the supernatant was removed. After one freeze–thaw cycle at −80 °C and 37 °C, the pellet was resuspended in 200 μL of 10 mg·mL^−1^ lysozyme in TE buffer. The mixture was incubated in a heat block at 37 °C for 5 min with vortexing every 2 min. Similarly, after one freeze–thaw cycle at −80 °C and 37 °C, the mixture was incubated in a heat block at 37 °C for 10 min with vortexing every 2 min. In addition, the mixture was subjected to five freeze–thaw cycles at −80 °C and 37 °C. Total RNA extraction was performed using the RNeasy Mini Kit (Qiagen) with on‐column DNase digestion which was performed using the RNase‐Free DNase Set (Qiagen) according to the manufacturer's instructions. Total RNA purification was performed in triplicate for each strain. Total RNA concentration was quantified using a GeneQuant *pro* (GE Healthcare, Chicago, IL, USA), and the 260/280 and 260/230 ratios were examined for protein and solvent contamination. Reverse transcription was performed on 500 ng of extracted total RNA using ReverTra Ace qPCR RT Master Mix with gDNA Remove (Toyobo, Osaka, Japan) according to the kit instructions.

### Quantitative real‐time PCR

Real‐time PCR was performed with THUNDERBIRD SYBR qPCR Mix (Toyobo) using AriaMx (Agilent, Santa Clara, CA, USA). Primers used in this study are described in Table 3 [31, 32]. Prior to qPCR, the amplification efficiency of the primers was tested. The reaction mixture was prepared as follows: 10 μL of THUNDERBIRD SYBR qPCR Mix, 0.6 μL of 10 μm Fw primer, 0.6 μL of 10 μm Rv primer, 4 μL of cDNA, and 4.8 μL of sterile distilled water for a total of 20 μL. Real‐time PCR was performed at 95 °C for 3 min, followed by 40 cycles at 95 °C for 15 s and 60 °C for 1 min. After the above reaction, melting curve analysis was performed at 95 °C for 30 s, 65 °C for 30 s, and 95 °C for 30 s to confirm that there was no non‐specific amplification. Each sample was measured in duplicate. *cysG* and *idnT* were used as reference genes [32], and the comparison of gene expression was evaluated using the ΔΔ*C*_t_ method [33].

**Table 3:** Primers used for quantitative real‐time PCR.

Gene	Primers	Sequences (5′–3′)	Efficiency	References
*bacA*	Fw	TGGGTGTGGTCGAAGGATTG	103.48	This work
	Rv	AAACCCCAACAAGTGACCGA		
*pgpB*	Fw	GTGTCTGCGTTTTCGCATTA	97.89	[31]
	Rv	ACAAAAGGTCGTGGTTCCTG		
*ybjG*	Fw	GCCGTGGTACTTTGGTTGTG	100.87	This work
	Rv	CGGTCGTGCGGAAAAAGATG		
*ispU*	Fw	CTGTCTCTTTTGCGGCCAAC	99.36	This work
	Rv	GCTGGTCGGTTCCAGTTTTC		
*cysG*	Fw	TTGTCGGCGGTGGTGATGTC	91.06	[32]
	Rv	ATGCGGTGAACTGTGGAATAAACG		
*idnT*	Fw	CTGTTTAGCGAAGAGGAGATGC	95.74	[32]
	Rv	ACAAACGGCGGCGATAGC		

### Statistical analysis

Statistical analysis was performed by one‐way ANOVA using Dunnett's multiple comparison test in the software, ezr [34].

## Results

### Quantification of C_55_P, C_55_PP, and total C_55_P derivatives

**Fig. 3:**
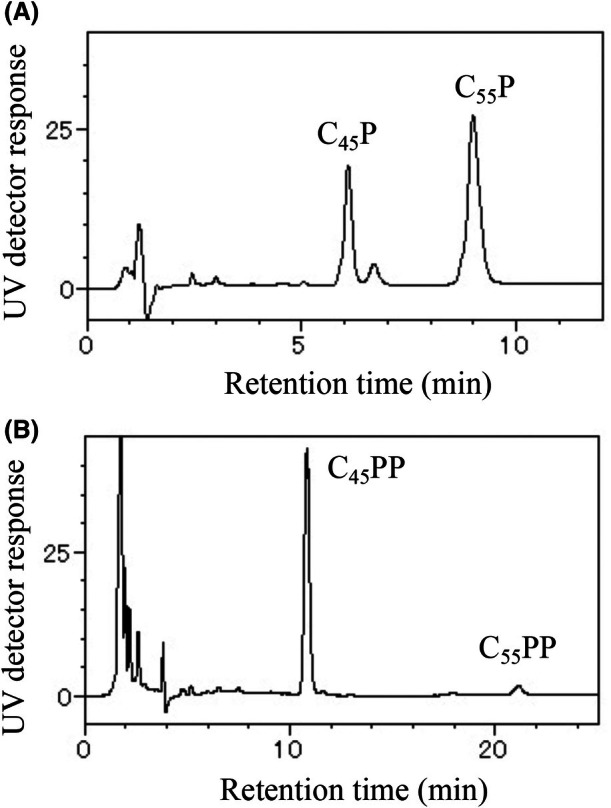
HPLC analysis of the polyprenyl phosphate and polyprenyl diphosphate from W3110. Polyprenyl phosphates and polyprenyl diphosphates were extracted from three biologically independent samples of all strains, separated by ion‐exchange chromatography, and analyzed by HPLC. Representative chromatograms are shown here. (A) Polyprenyl phosphate fraction. The sample was applied to a Mightysil RP‐18 GP Aqua column, and elution was carried out with 2‐propanol–methanol (1 : 4, v/v) containing 5 mm phosphoric acid. (B) Polyprenyl diphosphate fraction. The sample was applied to a Cadenza CD‐C18 MF column, and elution was carried out with 25 mm aqueous tetraethylammonium phosphate (pH 7.5)–2‐propanol (30 : 70, v/v).

**Fig. 4:**
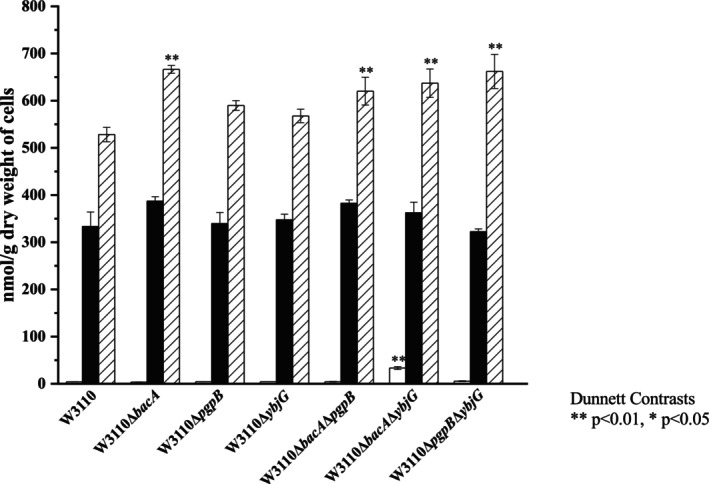
Levels of C_55_PP, C_55_P, and total C_55_P derivatives. Three biologically independent samples of all strains were analyzed. C_55_P and C_55_PP were extracted and then analyzed by HPLC. Total C_55_P derivatives were extracted from cells that were previously treated with alkali and then analyzed by HPLC. Statistical data are presented as the mean ± standard deviation (*n* = 3). White bar, C_55_PP; black bar, C_55_P; hatched bar, total C_55_P derivatives. Statistical significance by Dunnett's multiple comparison test is indicated (*P < 0.05 and **P < 0.01 compared with W3110).

The contents of C_55_P and C_55_PP in the *E. coli* cells grown to the exponential growth phase were determined by analyzing the lipids extracted with chloroform and methanol. The chloroform–methanol extract was passed through an anion exchange cartridge to obtain the polyprenyl phosphate fraction and the polyprenyl diphosphate fraction, which were then analyzed by HPLC to obtain the C_55_P (Fig. 3A) and C_55_PP (Fig. 3B) contents, respectively. The levels of C_55_P and C_55_PP in the wild‐type strain, W3110, were 333 ± 31 and 4.0 ± 0.2 nmol·g^−1^ dry weight of cells, respectively (Fig. 4). The total contents of C_55_P derivatives, including C_55_P, C_55_PP, and C_55_P‐linked and C_55_PP‐linked sugar intermediates, in the cells were determined by analyzing the lipids extracted with diethyl ether after treatment of the cells with alkali. Since C_55_PP and the C_55_P‐linked and C_55_PP‐linked sugar intermediates are hydrolyzed to C_55_P by alkaline treatment, the total content of C_55_P, C_55_PP, and the C_55_P‐linked and C_55_PP‐linked sugar intermediates could be determined by quantifying the C_55_P in the diethyl ether extract after alkaline treatment using HPLC. The total level of C_55_P derivatives in W3110 was 528 ± 15 nmol·g^−1^ dry weight of cells (Fig. 4). The levels of C_55_P and C_55_PP were 63% and 0.8% of the total level of C_55_P derivatives, respectively, indicating that the amount of C_55_PP was relatively low. The difference between the total level of C_55_P derivatives and the sum of levels of C_55_P and C_55_PP is considered to be the total level of various C_55_P‐linked and C_55_PP‐linked sugar intermediates, which accounted for 36% of the total level of C_55_P derivatives.

Next, we compared the contents of C_55_P, C_55_PP, and total C_55_P derivatives in the gene‐disruption strains. The levels of C_55_P were not significantly different from the wild‐type strain in any of the gene‐disruption strains. The level of C_55_PP was 33 ± 3 nmol·g^−1^ dry weight of cells in the *bacA* and *ybjG* double‐disruption strain, which was significantly higher than the level in the wild‐type strain (8.3 times higher). In other gene‐disruption strains, there was no significant difference in the level of C_55_PP compared to the wild‐type strain. The total levels of C_55_P derivatives were significantly higher in the *bacA* disruption strain and all double‐disruption strains than in the wild‐type strain, at approximately 1.3 times the level in the wild‐type strain.

### Bacitracin susceptibility testing

**Fig. 5:**
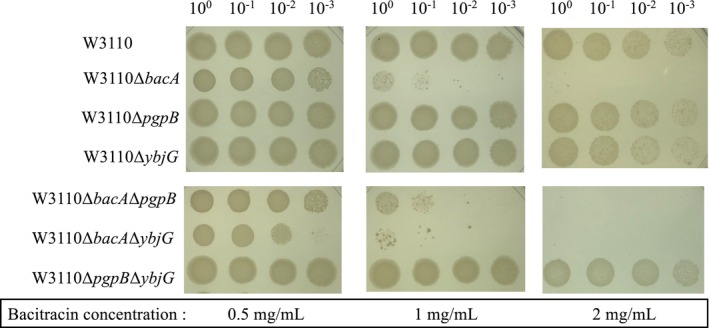
Bacitracin sensitivity. The overnight culture broth was normalized to OD_600_ = 1, and 10‐fold serial dilutions were prepared. Five microliter of each dilution was spotted onto L‐broth agar containing bacitracin at concentrations ranging between 0.5 and 2 mg·mL^−1^. Plates were incubated at 37 °C for 24 h. Three independent experiments were performed, and the results were consistent. The images shown are representative of the results obtained.

Bacitracin exerts its antibacterial effect by binding to C_55_PP and inhibiting the dephosphorylation of C_55_PP [19]. We examined the susceptibility of gene‐disruption strains to bacitracin as an indicator of the decrease in C_55_PP dephosphorylation activity in these strains (Fig. 5). The double‐disruption strain of *bacA* and *ybjG* was the most susceptible to bacitracin. The *bacA* disruption strain and *bacA*, *pgpB* double‐disruption strain showed the next highest susceptibility to bacitracin. The susceptibility to bacitracin of the *pgpB* disruption strain, *ybjG* disruption strain, and *pgpB*, *ybjG* double‐disruption strain was similar to that of the wild‐type strain.

### Expression analysis of genes involved in C_55_PP synthesis and dephosphorylation

**Fig. 6:**
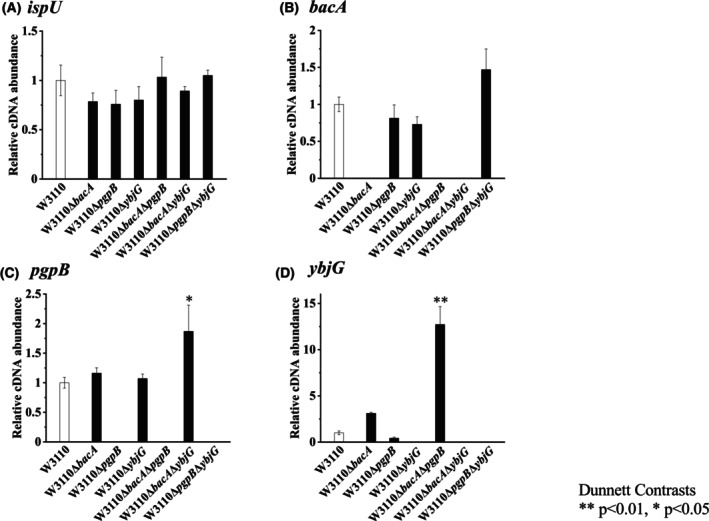
Transcriptional analysis of C_55_P synthesis‐related genes. The transcription of *ispU* (A), *bacA* (B), *pgpB* (C), and *ybjG* (D) genes was measured using three biologically independent samples for all strains. The wild‐type strain W3110 was normalized and compared with the gene‐disruption strains. Each qPCR measurement was performed in duplicate. Statistical data are presented as the mean ± standard deviation (*n* = 3). Statistical significance by Dunnett's multiple comparison test is indicated (*P < 0.05 and **P < 0.01 compared with W3110).

The level of C_55_P was not significantly changed by the disruption of the lipid phosphatase gene. To investigate whether the expression of genes involved in C_55_PP synthesis and dephosphorylation is regulated, the expression of the C_55_PP synthase gene, *ispU*, and the genes involved in the dephosphorylation of C_55_PP, *bacA*, *pgpB*, and *ybjG* were measured (Fig. 6). The expression of *ispU* in all disrupted strains was similar to that in the wild‐type strain (Fig. 6A). In the single‐disruption strains of the genes for C_55_PP dephosphorylation, the expression of the remaining genes was similar to that in the wild‐type strain (Fig. 6B–D). In the double‐disruption strains of the genes for C_55_PP dephosphorylation, the expression of the remaining genes tended to be higher than that in the wild‐type strain (Fig. 6B–D). The expression of *pgpB* in the *bacA*, *ybjG* double‐disruption strain was 1.8 times that of the wild‐type strain (Fig. 6C). The expression of *ybjG* in the double‐disruption strain of *bacA* and *pgpB* was 12.7 times higher than that of the wild‐type strain (Fig. 6D).

## Discussion

In *E. coli*, four enzymes, BacA, PgpB, YbjG, and LpxT, are responsible for the dephosphorylation of C_55_PP, which is involved in the supply of C_55_P [15]. It is predicted that the C_55_PP dephosphorylation activity will be lower in the gene‐disruption strains of these enzymes than in the wild‐type strain. In this study, we investigated how disrupting one or two of three genes, *bacA*, *pgpB*, and *ybjG*, affected the levels of C_55_PP and C_55_P. The level of C_55_PP, the substrate, in the single‐gene‐disruption strains was similar to that in the wild‐type strain. Among the double‐disruption strains, only the level of C_55_PP in the *bacA*, *ybjG* double‐disruption strain was significantly higher than that in the wild‐type strain at 8.3 times. The level of C_55_P, the product of the enzyme reaction, was similar in all strains. It was found that the remaining enzyme activity in the gene disruption strains is sufficient to provide C_55_P at the same level as the wild‐type strain.

The increased susceptibility to bacitracin is considered to be an indicator of the decreased intracellular C_55_PP dephosphorylation activity [15]. The fact that the bacitracin susceptibility of the *bacA* disruption strain and the double‐disruption strain containing the *bacA* disruption was higher than that of the wild‐type strain and other disruption strains suggests that the activity of BacA is higher than the total activity of the other two enzymes, PgpB and YbjG. This is consistent with the report by El Ghachi *et al*. [12], who reported that about 75% of the C_55_PP dephosphorylation activity measured *in vitro* is due to BacA activity. The double‐disruption strain of *bacA* and *ybjG* showed the highest susceptibility to bacitracin among the *bacA* disruption strains, indicating that it has the lowest C_55_PP dephosphorylation activity. This is consistent with the result that the C_55_PP level was highest in the double‐disruption strain of *bacA* and *ybjG*.

In gene‐disruption strains, the extent of the decrease in C_55_PP dephosphorylation activity should be reduced as the expression level of the remaining gene for C_55_PP dephosphorylation increases. In all double‐disruption strains, there was a tendency for the expression of the remaining single gene to increase compared to the wild‐type strain. Although there was no significant difference in the expression of *bacA* in the *pgpB*, *ybjG* double‐disruption strain compared to the wild‐type strain, the expression of *ybjG* and *pgpB* in the double‐disruption strains of *bacA* and *pgpB*, and *bacA* and *ybjG* was 12.7 and 1.8 times that of the wild‐type strain, respectively, and a significant difference was observed. The increase in gene expression in the double‐disruption strains is considered to contribute to the maintenance of the C_55_P level through the increase in intracellular C_55_PP dephosphorylation activity.

The levels of total C_55_P derivatives in the strains with *bacA* disruption and the strain with double disruption of *pgpB* and *ybjG* were significantly higher than those in the wild‐type strain, although the differences were small. The strains with *bacA* disruption and the strain with the double disruption of *pgpB* and *ybjG* were expected to have a significant decrease in intracellular C_55_PP dephosphorylation activity. It is possible that the synthesis of C_55_PP is increased to maintain the levels of C_55_P in these strains. Although there were no differences in the expression of the undecaprenyl diphosphate synthase gene, *ispU*, between the strains tested, it is possible that the stages of C_55_PP biosynthesis further upstream, such as the methylerythritol phosphate pathway of isopentenyl diphosphate synthesis [35], are regulated.

We found that the level of C_55_P is kept constant in all strains, while the expression of the lipid phosphatase genes, *pgpB* and *ybjG*, and the level of total C_55_P derivatives increase in some strains with gene disruption. It has been proposed that the control of C_55_P levels is important for normal bacterial growth from studies of mutant strains that accumulate C_55_PP‐linked sugar intermediates produced during the biosynthesis of exopolysaccharides [25, 26]. It is speculated that the activation of PAP2 enzyme gene transcription and increased isoprenoid biosynthesis in gene‐disruption strains contribute to the maintenance of C_55_P levels in these strains.

Finally, what is the mechanism underlying these regulations? First, a direct response to the decrease in C_55_PP dephosphorylation activity can be considered. Recently, Roney et al. have shown that the expression of genes involved in C_55_P supply in *Bacillus subtilis* is under the control of the transcription factor SigM, and they have proposed that the shortage of C_55_P is a direct signal to activate SigM [36]. On the other hand, it has been reported that the substrate C_55_PP inhibits the penicillin‐binding protein PBP1B, which is involved in peptidoglycan biosynthesis, and it is thought that the accumulation of C_55_PP is detrimental [37]. It would be reasonable to assume that the expression of the genes for C_55_PP dephosphorylation is regulated according to the level of C_55_P or C_55_PP, but it is difficult to say which is the case based on the results of this study. In the gene‐disruption strains, the total level of C_55_P derivatives increased, indicating increases in the level of C_55_P‐linked and C_55_PP‐linked sugar intermediates. Recently, examples have been reported in which the accumulation of specific C_55_PP‐linked sugar intermediates serves as a feedback regulatory signal [38]. Although a direct causal relationship between the decrease in C_55_PP dephosphorylation activity and the accumulation of any C_55_P‐linked or C_55_PP‐linked sugar intermediates is difficult to establish, it is possible that some C_55_P‐linked and C_55_PP‐linked sugar intermediates act as regulatory signals. Identification of the substance acting as a regulatory signal remains a future challenge.

## Conclusion

C_55_P levels were similar in the single‐ and double‐disruption strains of the phosphatase genes, *bacA*, *pgpB*, and *ybjG*, and the wild‐type strain. Transcription of the PAP2 family enzyme genes, *pgpB* and *ybjG*, was activated in the double‐disruption strains including *bacA* disruption, where C_55_PP dephosphorylation activity in cells was reduced. This transcriptional regulation may contribute to the maintenance of C_55_P levels in cells.

## Conflict of interest

The authors declare no conflict of interest.

## Peer review

The peer review history for this article is available at https://www.webofscience.com/api/gateway/wos/peer‐review/10.1002/2211‐5463.70066.

## Author contributions

TJ and SF conceived the study and designed the experiments. TJ performed the experiments and analyzed the data. YS supervised the analysis of compounds. TJ wrote the manuscript. YS and SF edited the manuscript. All authors reviewed and approved the final manuscript.

## Data Availability

The data that support the findings of this study are available from the corresponding author (sfujisak@biomol.sci.toho-u.ac.jp) upon reasonable request.

## References

[1] Galinier A , Delan‐Forino C , Foulquier E , Lakhal H and Pompeo F (2023) Recent advances in peptidoglycan synthesis and regulation in bacteria. Biomolecules 13, 720. 37238589 10.3390/biom13050720 PMC10216114

[2] Samuel G and Reeves P (2003) Biosynthesis of O‐antigens: genes and pathways involved in nucleotide sugar precursor synthesis and O‐antigen assembly. Carbohydr Res 338, 2503–2519. 14670712 10.1016/j.carres.2003.07.009

[3] Meier‐Dieter U , Starman R , Barr K , Mayer H and Rick PD (1990) Biosynthesis of enterobacterial common antigen in *Escherichia coli*. Biochemical characterization of Tn10 insertion mutants defective in enterobacterial common antigen synthesis. J Biol Chem 265, 13490–13497. 2166030

[4] Manat G , Roure S , Auger R , Bouhss A , Barreteau H , Mengin‐Lecreulx D and Touzé T (2014) Deciphering the metabolism of undecaprenyl‐phosphate: the bacterial cell‐wall unit carrier at the membrane frontier. Microb Drug Resist 20, 199–214. 24799078 10.1089/mdr.2014.0035 PMC4050452

[5] Kawakami N and Fujisaki S (2018) Undecaprenyl phosphate metabolism in Gram‐negative and Gram‐positive bacteria. Biosci Biotechnol Biochem 82, 940–946. 29198165 10.1080/09168451.2017.1401915

[6] Workman SD and Strynadka NCJ (2020) A slippery scaffold: synthesis and recycling of the bacterial cell wall carrier lipid. J Mol Biol 432, 4964–4982. 32234311 10.1016/j.jmb.2020.03.025

[7] Desai J , Wang Y , Wang K , Malwal SR and Oldfield E (2016) Isoprenoid biosynthesis inhibitors targeting bacterial cell growth. ChemMedChem 11, 2205–2215. 27571880 10.1002/cmdc.201600343 PMC5160999

[8] Apfel CM , Takács B , Fountoulakis M , Stieger M and Keck W (1999) Use of genomics to identify bacterial undecaprenyl pyrophosphate synthetase: cloning, expression, and characterization of the essential *uppS* gene. J Bacteriol 181, 483–492. 9882662 10.1128/jb.181.2.483-492.1999 PMC93402

[9] Kato J , Fujisaki S , Nakajima K , Nishimura Y , Sato M and Nakano A (1999) The *Escherichia coli* homologue of yeast Rer2, a key enzyme of dolichol synthesis, is essential for carrier lipid formation in bacterial cell wall synthesis. J Bacteriol 181, 2733–2738. 10217761 10.1128/jb.181.9.2733-2738.1999 PMC93712

[10] Rick PD , Barr K , Sankaran K , Kajimura J , Rush JS and Waechter CJ (2003) Evidence that the wxzE gene of *Escherichia coli* K‐12 encodes a protein involved in the transbilayer movement of a trisaccharide‐lipid intermediate in the assembly of enterobacterial common antigen. J Biol Chem 278, 16534–16542. 12621029 10.1074/jbc.M301750200

[11] Sham L‐T , Butler EK , Lebar MD , Kahne D , Bernhardt TG and Ruiz N (2014) MurJ is the flippase of lipid‐linked precursors for peptidoglycan biogenesis. Science 345, 220–222. 25013077 10.1126/science.1254522 PMC4163187

[12] El Ghachi M , Bouhss A , Blanot D and Mengin‐Lecreulx D (2004) The *bacA* gene of *Escherichia coli* encodes an undecaprenyl pyrophosphate phosphatase activity. J Biol Chem 279, 30106–30113. 15138271 10.1074/jbc.M401701200

[13] Roney IJ and Rudner DZ (2023) Two broadly conserved families of polyprenyl‐phosphate transporters. Nature 613, 729–734. 36450357 10.1038/s41586-022-05587-z PMC10184681

[14] Sit B , Srisuknimit V , Bueno E , Zingl FG , Hullahalli K , Cava F and Waldor MK (2023) Undecaprenyl phosphate translocases confer conditional microbial fitness. Nature 613, 721–728. 36450355 10.1038/s41586-022-05569-1 PMC9876793

[15] El Ghachi M , Derbise A , Bouhss A and Mengin‐Lecreulx D (2005) Identification of multiple genes encoding membrane proteins with undecaprenyl pyrophosphate phosphatase (UppP) activity in *Escherichia coli* . J Biol Chem 280, 18689–18695. 15778224 10.1074/jbc.M412277200

[16] Radeck J , Lautenschläger N and Mascher T (2017) The essential UPP phosphatase pair BcrC and UppP connects cell wall homeostasis during growth and sporulation with cell envelope stress response in *Bacillus subtilis* . Front Microbiol 8, 2403. 29259598 10.3389/fmicb.2017.02403 PMC5723303

[17] Touzé T , Tran AX , Hankins JV , Mengin‐Lecreulx D and Trent MS (2008) Periplasmic phosphorylation of lipid A is linked to the synthesis of undecaprenyl phosphate. Mol Microbiol 67, 264–277. 18047581 10.1111/j.1365-2958.2007.06044.x PMC2229476

[18] Cain BD , Norton PJ , Eubanks W , Nick HS and Allen CM (1993) Amplification of the *bacA* gene confers bacitracin resistance to *Escherichia coli* . J Bacteriol 175, 3784–3789. 8389741 10.1128/jb.175.12.3784-3789.1993 PMC204795

[19] Economou NJ , Cocklin S and Loll PJ (2013) High‐resolution crystal structure reveals molecular details of target recognition by bacitracin. Proc Natl Acad Sci USA 110, 14207–14212. 23940351 10.1073/pnas.1308268110 PMC3761639

[20] Chang HY , Chou CC , Hsu MF and Wang AH (2014) Proposed carrier lipid‐binding site of undecaprenyl pyrophosphate phosphatase from *Escherichia coli* . J Biol Chem 289, 18719–18735. 24855653 10.1074/jbc.M114.575076 PMC4081917

[21] Manat G , El Ghachi M , Auger R , Baouche K , Olatunji S , Kerff F , Touzé T , Mengin‐Lecreulx D and Bouhss A (2015) Membrane topology and biochemical characterization of the *Escherichia coli* BacA undecaprenyl‐pyrophosphate phosphatase. PLoS One 10, e0142870. 26560897 10.1371/journal.pone.0142870 PMC4641660

[22] Touzé T , Blanot D and Mengin‐Lecreulx D (2008) Substrate specificity and membrane topology of *Escherichia coli* PgpB, an undecaprenyl pyrophosphate phosphatase. J Biol Chem 283, 16573–16583. 18411271 10.1074/jbc.M800394200

[23] Tatar LD , Marolda CL , Polischuk AN , van Leeuwen D and Valvano MA (2007) An *Escherichia coli* undecaprenyl‐pyrophosphate phosphatase implicated in undecaprenyl phosphate recycling. Microbiology 153, 2518–2529. 17660416 10.1099/mic.0.2007/006312-0

[24] Fan J , Jiang D , Zhao Y , Liu J and Zhang XC (2014) Crystal structure of lipid phosphatase *Escherichia coli* phosphatidylglycerophosphate phosphatase B. Proc Natl Acad Sci USA 111, 7636–7640. 24821770 10.1073/pnas.1403097111 PMC4040569

[25] Jorgenson MA and Young KD (2016) Interrupting biosynthesis of O antigen or the lipopolysaccharide core produces morphological defects in *Escherichia coli* by sequestering undecaprenyl phosphate. J Bacteriol 198, 3070–3079. 27573014 10.1128/JB.00550-16 PMC5075036

[26] Jorgenson MA , Kannan S , Laubacher ME and Young KD (2016) Dead‐end intermediates in the enterobacterial common antigen pathway induce morphological defects in *Escherichia coli* by competing for undecaprenyl phosphate. Mol Microbiol 100, 1–14. 26593043 10.1111/mmi.13284 PMC4845916

[27] Jitsukawa T , Watanabe S , Shigeri Y and Fujisaki S (2024) Quantification of polyprenyl diphosphates in *Escherichia coli* cells using high‐performance liquid chromatography. Biosci Biotechnol Biochem 88, 429–436. 38192035 10.1093/bbb/zbae001

[28] Saito Y , Ishikawa T , Murakami M , Suzuki K and Fujisaki S (2013) Mutant deletions in *Escherichia coli* affect the cellular levels of undecaprenyl phosphate and undecaprenyl diphosphate. J Biol Macromol 13, 86–91.

[29] Baba T , Ara T , Hasegawa M , Takai Y , Okumura Y , Baba M , Datsenko KA , Tomita M , Wanner BL and Mori H (2006) Construction of *Escherichia coli* K‐12 in‐frame, single‐gene knockout mutants: the Keio collection. Mol Syst Biol 2, 2006.0008. 10.1038/msb4100050 PMC1681482 16738554

[30] Datsenko KA and Wanner BL (2000) One‐step inactivation of chromosomal genes in *Escherichia coli* K‐12 using PCR products. Proc Natl Acad Sci USA 97, 6640–6645. 10829079 10.1073/pnas.120163297 PMC18686

[31] Tian X , Auger R , Manat G , Kerff F , Mengin‐Lecreulx D and Touzé T (2020) Insight into the dual function of lipid phosphate phosphatase PgpB involved in two essential cell‐envelope metabolic pathways in *Escherichia coli* . Sci Rep 10, 13209. 32764655 10.1038/s41598-020-70047-5 PMC7413402

[32] Zhou K , Zhou L , Lim Q , Zou R , Stephanopoulos G and Too HP (2011) Novel reference genes for quantifying transcriptional responses of *Escherichia coli* to protein overexpression by quantitative PCR. BMC Mol Biol 12, 18. 21513543 10.1186/1471-2199-12-18 PMC3110127

[33] Schmittgen TD and Livak KJ (2008) Analyzing real‐time PCR data by the comparative C(T) method. Nat Protoc 3, 1101–1108. 18546601 10.1038/nprot.2008.73

[34] Kanda Y (2013) Investigation of the freely available easy‐to‐use software ‘EZR’ for medical statistics. Bone Marrow Transplant 48, 452–458. 23208313 10.1038/bmt.2012.244 PMC3590441

[35] Martin VJJ , Pitera DJ , Withers ST , Newman JD and Keasling JD (2003) Engineering a mevalonate pathway in *Escherichia coli* for production of terpenoids. Nat Biotechnol 21, 796–802. 12778056 10.1038/nbt833

[36] Roney IJ and Rudner DZ (2024) *Bacillus subtilis* uses the SigM signaling pathway to prioritize the use of its lipid carrier for cell wall synthesis. PLoS Biol 22, e3002589. 38683856 10.1371/journal.pbio.3002589 PMC11081497

[37] Hernández‐Rocamora VM , Otten CF , Radkov A , Simorre JP , Breukink E , VanNieuwenhze M and Vollmer W (2018) Coupling of polymerase and carrier lipid phosphatase prevents product inhibition in peptidoglycan synthesis. Cell Surf 2, 1–13. 30046664 10.1016/j.tcsw.2018.04.002 PMC6053597

[38] Marmont LS , Orta AK , Baileeves BWA , Sychantha D , Fernández‐Galliano A , Li YE , Greene NG , Corey RA , Stansfeld PJ , Clemons WM Jr *et al*. (2024) Synthesis of lipid‐linked precursors of the bacterial cell wall is governed by a feedback control mechanism in *Pseudomonas aeruginosa* . Nat Microbiol 9, 763–775. 38336881 10.1038/s41564-024-01603-2 PMC10914600

